# Supplementary material for: An open-source framework for large-scale, flexible evaluation of biomedical text mining systems
Source: J Biomed Discov Collab. 2008 Jan 29;3:1. doi: 10.1186/1747-5333-3-1 (PMC2276192; doi:10.1186/1747-5333-3-1)
Supplement: Additional file 1 — Evaluation platform source code. The additional file contains the software evaluation framework discussed in this paper. The most current release can be downloaded from SourceForge.net [1]. See the accompanying README file for instructions on installation and use. Also included in the distribution is a collection of UIMA wrappers for some commonly used BioNLP tools and annotated corpora. [file 1747-5333-3-1-S1.zip › bionlp_uima_repository-1.0.1/docs/api/src-html/edu/uchsc/ccp/uima/cc/summary/comparison/AnnotationComparator_CC.html]

```
001    /*
002     * Created on Apr 19, 2005
003     * Revised 6/9/06 to comply with updated type system and revised NLPStats
004     * Revised 3/29/07 to comply with new annotation comparison machinery
005     * 
006     */
007    package edu.uchsc.ccp.uima.cc.summary.comparison;
008    
009    import java.io.IOException;
010    import java.io.PrintStream;
011    import java.util.ArrayList;
012    import java.util.HashMap;
013    import java.util.Iterator;
014    import java.util.List;
015    import java.util.Map;
016    import java.util.Set;
017    
018    import org.apache.uima.cas.CAS;
019    import org.apache.uima.cas.CASException;
020    import org.apache.uima.collection.CasConsumer_ImplBase;
021    import org.apache.uima.collection.base_cpm.CasObjectProcessor;
022    import org.apache.uima.jcas.JCas;
023    import org.apache.uima.resource.ResourceInitializationException;
024    import org.apache.uima.resource.ResourceProcessException;
025    import org.apache.uima.util.ProcessTrace;
026    
027    import edu.uchsc.ccp.uima.annotation.CCPDocumentInformation;
028    import edu.uchsc.ccp.uima.util.UIMA_Util;
029    import edu.uchsc.ccp.util.nlp.GenericTextUtils;
030    import edu.uchsc.ccp.util.nlp.annotation.TextAnnotation;
031    import edu.uchsc.ccp.util.nlp.annotation.comparison.AnnotationComparator;
032    import edu.uchsc.ccp.util.nlp.annotation.comparison.IgnoreSpanComparator;
033    import edu.uchsc.ccp.util.nlp.annotation.comparison.SharedEndSpanComparator;
034    import edu.uchsc.ccp.util.nlp.annotation.comparison.SharedStartOrEndSpanComparator;
035    import edu.uchsc.ccp.util.nlp.annotation.comparison.SharedStartSpanComparator;
036    import edu.uchsc.ccp.util.nlp.annotation.comparison.SloppySpanComparator;
037    import edu.uchsc.ccp.util.nlp.annotation.comparison.SpanComparator;
038    import edu.uchsc.ccp.util.nlp.annotation.comparison.StrictSpanComparator;
039    import edu.uchsc.ccp.util.nlp.mention.comparison.ClassMentionComparator;
040    import edu.uchsc.ccp.util.nlp.mention.comparison.IdenticalClassMentionComparator;
041    import edu.uchsc.ccp.util.nlp.stats.PRFResult;
042    
043    /**
044     * @author williamb
045     * 
046     */
047    public class AnnotationComparator_CC extends CasConsumer_ImplBase implements CasObjectProcessor {
048    
049        private final boolean DEBUG = false;
050    
051        private SpanComparator spanComparator;
052    
053        private ClassMentionComparator mentionComparator;
054    
055        private String spanComparatorUsed = "";
056    
057        private String mentionComparatorUsed = "";
058    
059        public static final String PARAM_CONFIG_FILE = "ConfigFile";
060    
061        /* span comparison strategies */
062        public static final String PARAM_USE_STRICT_SPAN_COMPARATOR = "UseStrictSpanComparator";
063    
064        public static final String PARAM_USE_OVERLAP_SPAN_COMPARATOR = "UseOverlapSpanComparator";
065    
066        public static final String PARAM_USE_SHARED_START_SPAN_COMPARATOR = "UseSharedStartSpanComparator";
067    
068        public static final String PARAM_USE_SHARED_END_SPAN_COMPARATOR = "UseSharedEndSpanComparator";
069    
070        public static final String PARAM_USE_SHARED_START_OR_END_SPAN_COMPARATOR = "UseSharedStartOrEndSpanComparator";
071    
072        public static final String PARAM_USE_IGNORE_SPAN_COMPARATOR = "UseIgnoreSpanComparator";
073        
074        /* mention comparison strategies */
075        public static final String PARAM_USE_IDENTICAL_MENTION_COMPARATOR = "UseIdenticalMentionComparator";
076        
077        
078    
079        /**
080         * if filled, TP, FP, and FN annotations will be printed to file */
081        public static final String PARAM_ANNOTATION_OUTPUT_FILE = "AnnotationOutputFile";
082        
083        private PrintStream outputStream = null;
084        
085        /* hash for mapping annotations to groupings */
086        private HashMap<String, Integer> groupingsMap;
087    
088        private int goldStandardIndex = -1;
089    
090        // private List<Map<String, PRFResult>> scores;
091        private List<PRFResult> scores;
092    
093        private List<ComparisonGroup> comparisonGroupList;
094    
095        /**
096         * Initializes this CAS Consumer with the parameters specified in the descriptor.
097         * 
098         * @throws ResourceInitializationException
099         *             if there is error in initializing the resources
100         */
101        public void initialize() throws ResourceInitializationException {
102            
103    
104            /* read in input parameters */
105            String configFile = (String) getConfigParameterValue(PARAM_CONFIG_FILE);
106            /* Span comparison metrics */
107            Boolean useStrictSpanComparator = (Boolean) getConfigParameterValue(PARAM_USE_STRICT_SPAN_COMPARATOR);
108            Boolean useOverlapSpanComparator = (Boolean) getConfigParameterValue(PARAM_USE_OVERLAP_SPAN_COMPARATOR);
109            Boolean useSharedStartSpanComparator = (Boolean) getConfigParameterValue(PARAM_USE_SHARED_START_SPAN_COMPARATOR);
110            Boolean useSharedEndSpanComparator = (Boolean) getConfigParameterValue(PARAM_USE_SHARED_END_SPAN_COMPARATOR);
111            Boolean useSharedStartOrEndSpanComparator = (Boolean) getConfigParameterValue(PARAM_USE_SHARED_START_OR_END_SPAN_COMPARATOR);
112            Boolean useIgnoreSpanComparator = (Boolean) getConfigParameterValue(PARAM_USE_IGNORE_SPAN_COMPARATOR);
113            
114            /* Mention comparison metrics */
115            Boolean useIdenticalMentionComparator = (Boolean) getConfigParameterValue(PARAM_USE_IDENTICAL_MENTION_COMPARATOR);
116    
117            /* Retrieve output file if there is one */
118            String outputFile = (String) getConfigParameterValue(PARAM_ANNOTATION_OUTPUT_FILE);
119            if (outputFile != null) {
120                try {
121                    outputStream = GenericTextUtils.openPrintStream(outputFile);
122                } catch (IOException e) {
123                    e.printStackTrace();
124                }
125            }
126            
127            /* Parse the configuration file */
128            System.err.println("Initializing AnnotationComparator... loading config file: " + configFile);
129            ComparatorConfigurator configurator = new ComparatorConfigurator(configFile);
130            Map<AnnotationGroupID, AnnotationGroup> annotationGroupMap = configurator.getAnnotationGroupMap();
131            comparisonGroupList = configurator.getComparisonGroupList();
132    
133            /* Determine gold standard index */
134            for (int i = 0; i < comparisonGroupList.size(); i++) {
135                if (comparisonGroupList.get(i).isGoldStandard()) {
136                    goldStandardIndex = i;
137                }
138            }
139    
140            /* check to see that there was a gold standard group provided */
141            if (goldStandardIndex == -1) {
142                warn("No Gold Standard ComparisonGroup provided. By default, the first comparison group will be used as the gold standard.");
143                goldStandardIndex = 0;
144            }
145    
146            /*
147             * create hash for mapping annotations to ComparisonGroups -- hash key is [annotationSetID annotatorID conceptMentionType], hash value is the
148             * index in the comparisonGroupList
149             */
150            groupingsMap = new HashMap<String, Integer>();
151            scores = new ArrayList<PRFResult>();
152    
153            for (int i = 0; i < comparisonGroupList.size(); i++) {
154                if (DEBUG) {
155                    comparisonGroupList.get(i).print(System.err);
156                }
157                /* for each comparison group, get the annotation groups that belong to it */
158                List<AnnotationGroupID> annotationGroups = comparisonGroupList.get(i).getAnnotationGroupList();
159                for (AnnotationGroupID agID : annotationGroups) {
160                    /* get the annotation group, and retrieve the annotationID, annotatorID, and annotationTypes */
161                    AnnotationGroup aGroup = annotationGroupMap.get(agID);
162                    if (DEBUG) {
163                        aGroup.print(System.err);
164                    }
165                    Integer asetID = aGroup.getAnnotationSetID();
166                    Integer aID = aGroup.getAnnotatorID();
167                    for (String type : aGroup.getAnnotationTypeList()) {
168                        String annotationKey = asetID + " " + aID + " " + type;
169                        if (groupingsMap.containsKey(annotationKey)) {
170                            warn("Attempted duplicate Annotation Group. Key: '" + annotationKey + "' ('AnnotationSetID AnnotatorID ClassType') has already been assigned to a group. Please check the Annotation Comparator config file.");
171                        } else {
172                            groupingsMap.put(annotationKey, new Integer(i));
173                        }
174                    }
175                }
176    
177                /* initialize a new PRFResult that will hold the score for that group */
178                scores.add(new PRFResult(0, 0, 0));
179            }
180    
181            /* Initialize the SpanComparator and MentionComparator that will be used */
182            if (useStrictSpanComparator != null && useStrictSpanComparator) {
183                spanComparator = new StrictSpanComparator();
184            } else if (useOverlapSpanComparator != null && useOverlapSpanComparator) {
185                spanComparator = new SloppySpanComparator();
186            } else if (useSharedStartSpanComparator != null && useSharedStartSpanComparator) {
187                spanComparator = new SharedStartSpanComparator();
188            } else if (useSharedEndSpanComparator != null && useSharedEndSpanComparator) {
189                spanComparator = new SharedEndSpanComparator();
190            } else if (useSharedStartOrEndSpanComparator != null && useSharedStartOrEndSpanComparator) {
191                spanComparator = new SharedStartOrEndSpanComparator();
192            } else if (useIgnoreSpanComparator != null && useIgnoreSpanComparator) {
193                spanComparator = new IgnoreSpanComparator();
194            } else {
195                warn("No SpanComparator chosen. Please check your CPE descriptor file. Using StrictSpanComparator by default.");
196                spanComparator = new StrictSpanComparator();
197            }
198    
199            if (useIdenticalMentionComparator != null && useIdenticalMentionComparator) {
200                mentionComparator = new IdenticalClassMentionComparator();
201            } else {
202                warn("No MentionComparator chosen. Please check your CPE descriptor file. Using IdenticalMentionComparator by default.");
203            }
204    
205            spanComparatorUsed = spanComparator.getClass().getName().substring(spanComparator.getClass().getName().lastIndexOf('.') + 1);
206            mentionComparatorUsed = mentionComparator.getClass().getName().substring(
207                    mentionComparator.getClass().getName().lastIndexOf('.') + 1);
208    
209            super.initialize();
210        }
211    
212        public void processCas(CAS aCAS) throws ResourceProcessException {
213            JCas jcas;
214            try {
215                jcas = aCAS.getJCas();
216            } catch (CASException e) {
217                throw new ResourceProcessException(e);
218            }
219    
220            /* get the documentID */
221            String documentID = null;
222            Iterator docInfoIter = jcas.getJFSIndexRepository().getAnnotationIndex(CCPDocumentInformation.type).iterator();
223            if (docInfoIter.hasNext()) {
224                CCPDocumentInformation ccpDocInfo = (CCPDocumentInformation) docInfoIter.next();
225                documentID = ccpDocInfo.getDocumentID();
226            } else {
227                error("Expecting CCPDocumentInformation annotation, but did not find one.");
228                documentID = "-1";
229            }
230            
231            /* set up annotation storage by creating a list of TextAnnotationLists */
232            List<List<TextAnnotation>> annotationsToCompare = new ArrayList<List<TextAnnotation>>();
233            for (int i = 0; i < comparisonGroupList.size(); i++) {
234                annotationsToCompare.add(new ArrayList<TextAnnotation>());
235            }
236    
237            /* get the TextAnnotations out of the CAS and put them in the proper comparison group(s) */
238            UIMA_Util uimaUtil = new UIMA_Util();
239            List<TextAnnotation> annotations = uimaUtil.getTextAnnotationsFromJCas(jcas);
240    
241            for (TextAnnotation ta : annotations) {
242                /* get the annotatorID */
243                Integer annotatorID = ta.getAnnotator().getAnnotatorID();
244                /* get the annotationSetID */
245                Set<Integer> aSetIDs = ta.getAnnotationSetIDs();
246                /* get the class mention type */
247                String classMentionType = ta.getClassMention().getMentionName();
248    
249                /* for each annotation set that this annotation is associated with, create a key and see if that key is in the groupingsMap */
250                for (Integer aSetID : aSetIDs) {
251                    String key = aSetID + " " + annotatorID + " " + classMentionType;
252                    debug("Key: " + key);
253                    if (classMentionType != null) {
254                        /* if the current annotation matches a hash key, then store the annotation in the appropriate group */
255                        if (groupingsMap.containsKey(key)) {
256                            int group = groupingsMap.get(key).intValue();
257                            debug("Storing Key: " + key + " in group: " + group);
258                            annotationsToCompare.get(group).add(ta);
259                        }
260                    } else {
261                        warn("Null class mention type discovered...");
262                        ta.printAnnotation(System.err);
263                    }
264                }
265            }
266    
267            if (DEBUG) {
268                /* print out the groups of annotations that will be compared */
269                int index = 0;
270                for (List<TextAnnotation> taList : annotationsToCompare) {
271                    debug("%%%%%%%%%%%%%%%%%%%%%%%% COMPARISON GROUP: " + index++ + " %%%%%%%%%%%%%%%%%%%%%%%%%%%%%%% GoldStandard Index = " + goldStandardIndex);
272                    for (TextAnnotation ta : taList) {
273                        ta.printAnnotation(System.err);
274                    }
275                }
276            }
277            
278            
279            AnnotationComparator annotationComparator = new AnnotationComparator();
280    
281            /* do annotation comparisons */
282    
283            for (int i = 0; i < annotationsToCompare.size(); i++) {
284                PRFResult prf = annotationComparator.compare(annotationsToCompare.get(goldStandardIndex), annotationsToCompare.get(i),
285                        spanComparator, mentionComparator);
286    
287                /* if we are outputting, then output the annotations for this document to file */
288                if (outputStream != null) {
289                    outputStream.println("################  DocumentID: " + documentID + "  ################");
290                    outputStream.println("Analysis of \"" + comparisonGroupList.get(i).getDescription() + "\" vs. \""
291                            + comparisonGroupList.get(goldStandardIndex).getDescription() + "\"");
292                    prf.printAnnotations(outputStream);
293                }
294                
295                /* update the total score with this incremental scores */
296                scores.get(i).add(prf);
297            }
298    
299        }
300    
301        /**
302         * Print the comparison results
303         */
304        public void collectionProcessComplete(ProcessTrace aTrace) throws ResourceProcessException, IOException {
305            super.collectionProcessComplete(aTrace);
306    
307            
308            PrintStream ps = System.out;
309    
310            for (int i = 0; i < comparisonGroupList.size(); i++) {
311                String outStr= "Analysis of \"" + comparisonGroupList.get(i).getDescription() + "\" vs. \""
312                        + comparisonGroupList.get(goldStandardIndex).getDescription() + "\"";
313                ps.println(outStr);
314                
315                PRFResult prf = scores.get(i);
316                prf.setTitle("SpanComparator: " + spanComparatorUsed + " -- MentionComparator: " + mentionComparatorUsed);
317                prf.printStats(ps);
318                
319                if (outputStream != null) {
320                    outputStream.println();
321                    outputStream.println(outStr);
322                    prf.printStats(outputStream);
323                }
324                
325            }
326            
327            /* close the output file if there is one */
328            if (outputStream != null) {
329                outputStream.close();
330            }
331        }
332    
333        // /**
334        // * Initializes this CAS Consumer with the parameters specified in the descriptor.
335        // *
336        // * @throws ResourceInitializationException
337        // * if there is error in initializing the resources
338        // */
339        // public void initialize() throws ResourceInitializationException {
340        // System.err.println("Initializing AnnotationComparator...");
341        //
342        // /* read in input parameters */
343        // String configFile = (String) getConfigParameterValue(PARAM_CONFIG_FILE);
344        //
345        // /* Parse the configuration file */
346        // ComparatorConfigurator configurator = new ComparatorConfigurator(configFile);
347        // Map<AnnotationGroupID, AnnotationGroup> annotationGroupMap = configurator.getAnnotationGroupMap();
348        // comparisonGroupList = configurator.getComparisonGroupList();
349        //
350        // /* Determine gold standard index */
351        // for (int i = 0; i < comparisonGroupList.size(); i++) {
352        // if (comparisonGroupList.get(i).isGoldStandard()) {
353        // goldStandardIndex = i;
354        // }
355        // }
356        //
357        // /* check to see that there was a gold standard group provided */
358        // if (goldStandardIndex == -1) {
359        // System.err
360        // .println("WARNING -- AnnotationComparator: No Gold Standard ComparisonGroup provided. By default, the first comparison group will be used as
361        // the gold standard.");
362        // goldStandardIndex = 0;
363        // }
364        //
365        // /*
366        // * set up score storage for the 6 metrics and n comparisons -- "STRICT", "SLOPPY", "PNP", "LEFT BOUNDARY", "RIGHT BOUNDARY", "EITHER
367        // * BOUNDARY" There are really n-1 comparisons, however we will compare the gold standard annotations against themselves as a
368        // * validity check.
369        // */
370        // scores = new ArrayList<Map<String, PRFStats>>();
371        // for (int i = 0; i < comparisonGroupList.size(); i++) {
372        // Map<String, PRFStats> scoreMap = new HashMap<String, PRFStats>();
373        // scoreMap.put(Annotation_Util.MATCH_STRICT, new PRFStats(0, 0, 0, "STRICT"));
374        // scoreMap.put(Annotation_Util.MATCH_SLOPPY, new PRFStats(0, 0, 0, "SLOPPY"));
375        // scoreMap.put(Annotation_Util.MATCH_PNP, new PRFStats(0, 0, 0, "PNP"));
376        // scoreMap.put(Annotation_Util.MATCH_BOUNDARY_LEFT, new PRFStats(0, 0, 0, "LEFT BOUNDARY"));
377        // scoreMap.put(Annotation_Util.MATCH_BOUNDARY_RIGHT, new PRFStats(0, 0, 0, "RIGHT BOUNDARY"));
378        // scoreMap.put(Annotation_Util.MATCH_BOUNDARY_EITHER, new PRFStats(0, 0, 0, "EITHER BOUNDARY"));
379        // scores.add(scoreMap);
380        // }
381        //
382        // /*
383        // * create hash for mapping annotations to ComparisonGroups -- hash key is [annotationSetID annotatorID conceptMentionType], hash
384        // * value is the index in the comparisonGroupList
385        // */
386        // groupingsMap = new HashMap<String, Integer>();
387        //
388        // for (int i = 0; i < comparisonGroupList.size(); i++) {
389        // if (DEBUG) {
390        // comparisonGroupList.get(i).print(System.err);
391        // }
392        // /* for each comparison group, get the annotation groups that belong to it */
393        // List<AnnotationGroupID> annotationGroups = comparisonGroupList.get(i).getAnnotationGroupList();
394        // for (AnnotationGroupID agID : annotationGroups) {
395        // /* get the annotation group, and retrieve the annotationID, annotatorID, and annotationTypes */
396        // AnnotationGroup aGroup = annotationGroupMap.get(agID);
397        // if (DEBUG) {
398        // aGroup.print(System.err);
399        // }
400        // AnnotationSetID asetID = aGroup.getAnnotationSetID();
401        // AnnotatorID aID = aGroup.getAnnotatorID();
402        // for (String type : aGroup.getAnnotationTypeList()) {
403        // String annotationKey = asetID + " " + aID + " " + type;
404        // groupingsMap.put(annotationKey, new Integer(i));
405        // }
406        // }
407        // }
408        //
409        // super.initialize();
410        // }
411    
412        // public void processCas(CAS aCAS) throws ResourceProcessException {
413        // JCas jcas;
414        // try {
415        // jcas = aCAS.getJCas();
416        // } catch (CASException e) {
417        // throw new ResourceProcessException(e);
418        // }
419        //
420        // if (DEBUG) {
421        // System.err.println("DEBUG -- AnnotationComparator: PROCESSING JCAS");
422        // }
423        //
424        // // /* Get the document ID */
425        // // String documentID = null;
426        // // Iterator docInfoIter = jcas.getJFSIndexRepository().getAnnotationIndex(CCPDocumentInformation.type).iterator();
427        // // if (docInfoIter.hasNext()) {
428        // // Object possibleCCPDocInfo = docInfoIter.next();
429        // // if (possibleCCPDocInfo instanceof CCPDocumentInformation) {
430        // // CCPDocumentInformation ccpDocInfo = (CCPDocumentInformation) possibleCCPDocInfo;
431        // // documentID = ccpDocInfo.getDocumentID();
432        // // } else {
433        // // warn("NO DOC INFO FOUND!!!");
434        // // }
435        // // }
436        //        
437        // /* set up annotation storage by creating a list of TextAnnotationLists */
438        // List<List<TextAnnotation>> annotationsToCompare = new ArrayList<List<TextAnnotation>>();
439        // for (int i = 0; i < comparisonGroupList.size(); i++) {
440        // annotationsToCompare.add(new ArrayList<TextAnnotation>());
441        // }
442        //
443        // /* get the TextAnnotations out of the CAS */
444        // UIMA_Util uimaUtil = new UIMA_Util();
445        // List<TextAnnotation> annotations = uimaUtil.getTextAnnotationsFromJCas(jcas);
446        //
447        //        
448        // // if (documentID.equals("38839")) {
449        // // System.out.println("+++++++++++++++++++++++++++++++++++++++++++++++++++++++++++++++++++++++++++++++++++++++++++++++++++");
450        // // System.out.println("INPUT ANNOTATIONS - ANNOTATION COMPARATOR");
451        // // System.out.println("+++++++++++++++++++++++++++++++++++++++++++++++++++++++++++++++++++++++++++++++++++++++++++++++++++");
452        // // /* print the input annotations */
453        // // for (TextAnnotation ta : annotations) {
454        // // ta.printAnnotation(System.out);
455        // // }
456        // // }
457        //        
458        // if (DEBUG) {
459        // System.err.println("DEBUG -- AnnotationComparator: # annotations in JCas: " + annotations.size());
460        // }
461        //        
462        // for (TextAnnotation ta : annotations) {
463        // /* get the annotatorID */
464        // Integer annotatorID = ta.getAnnotator().getAnnotatorID();
465        // /* get the annotationSetID */
466        // List<Integer> aSetIDs = ta.getAnnotationSetIDs();
467        // /* get the class mention type */
468        // String classMentionType = ta.getClassMention().getMentionName();
469        //
470        // /* for each annotation set that this annotation is associated with, create a key and see if that key is in the groupingsMap */
471        // for (Integer aSetID : aSetIDs) {
472        // String key = aSetID + " " + annotatorID + " " + classMentionType;
473        // if (DEBUG) {
474        // System.err.println("DEBUG -- AnnotationComparator: Checking KEY to see if we should add TA to a group: " + key);
475        // }
476        //
477        // if (classMentionType != null) {
478        // /* if the current annotation matches a hash key, then store the annotation in the appropriate group */
479        // if (groupingsMap.containsKey(key)) {
480        // if (DEBUG) {
481        // System.err.println("DEBUG -- AnnotationComparator: Adding TextAnnotation to group...");
482        // }
483        // int group = groupingsMap.get(key).intValue();
484        // annotationsToCompare.get(group).add(ta);
485        // }
486        // } else {
487        // System.err.println("WARNING -- AnnotationComparator: Null class mention type discovered...");
488        // ta.printAnnotation(System.err);
489        // }
490        // }
491        // }
492        //
493        // /* do annotation comparisons */
494        // PRFStats strictForDocument = new PRFStats(0,0,0,"STRICT MATCH OVER SINGLE DOCUMENT");
495        // for (int i = 0; i < annotationsToCompare.size(); i++) {
496        // PRFStats strictStats = Annotation_Util.matchAnnotations_STRICT(annotationsToCompare.get(goldStandardIndex),
497        // annotationsToCompare.get(i), false, true, "STRICT");
498        // PRFStats sloppyStats = Annotation_Util.matchAnnotations_SLOPPY(annotationsToCompare.get(goldStandardIndex),
499        // annotationsToCompare.get(i), false, true);
500        // PRFStats pnpStats = Annotation_Util.matchAnnotations_PNP(annotationsToCompare.get(goldStandardIndex), annotationsToCompare
501        // .get(i), false, true);
502        // PRFStats lbStats = Annotation_Util.matchAnnotations_BOUNDARY(annotationsToCompare.get(goldStandardIndex), annotationsToCompare
503        // .get(i), Annotation_Util.MATCH_BOUNDARY_LEFT, false, true);
504        // PRFStats rbStats = Annotation_Util.matchAnnotations_BOUNDARY(annotationsToCompare.get(goldStandardIndex), annotationsToCompare
505        // .get(i), Annotation_Util.MATCH_BOUNDARY_RIGHT, false, true);
506        // PRFStats ebStats = Annotation_Util.matchAnnotations_BOUNDARY(annotationsToCompare.get(goldStandardIndex), annotationsToCompare
507        // .get(i), Annotation_Util.MATCH_BOUNDARY_EITHER, false, true);
508        //
509        // if (DEBUG && i == 1) {
510        // strictStats.printStats(System.err);
511        // strictForDocument.add(strictStats);
512        // }
513        //            
514        // /* update the stored scores with these incremental scores */
515        // Map<String, PRFStats> scoreMap = scores.get(i);
516        // scoreMap.get(Annotation_Util.MATCH_STRICT).add(strictStats);
517        // scoreMap.get(Annotation_Util.MATCH_SLOPPY).add(sloppyStats);
518        // scoreMap.get(Annotation_Util.MATCH_PNP).add(pnpStats);
519        // scoreMap.get(Annotation_Util.MATCH_BOUNDARY_LEFT).add(lbStats);
520        // scoreMap.get(Annotation_Util.MATCH_BOUNDARY_RIGHT).add(rbStats);
521        // scoreMap.get(Annotation_Util.MATCH_BOUNDARY_EITHER).add(ebStats);
522        // }
523        //
524        // if (DEBUG) {
525        // strictForDocument.printStats(System.err);
526        // }
527        //        
528        //        
529        //        
530        // // if (documentID.equals("38839")) {
531        // // System.out.println("+++++++++++++++++++++++++++++++++++++++++++++++++++++++++++++++++++++++++++++++++++++++++++++++++++");
532        // // System.out.println("OUTPUT ANNOTATIONS - ANNOTATION COMPARATOR");
533        // // System.out.println("+++++++++++++++++++++++++++++++++++++++++++++++++++++++++++++++++++++++++++++++++++++++++++++++++++");
534        // // /* print the input annotations */
535        // // for (TextAnnotation ta : annotations) {
536        // // ta.printAnnotation(System.out);
537        // // }
538        // // }
539        //        
540        // }
541    
542        // /**
543        // * Print the comparison results
544        // */
545        // public void collectionProcessComplete(ProcessTrace aTrace) throws ResourceProcessException, IOException {
546        // super.collectionProcessComplete(aTrace);
547        //
548        // PrintStream ps = System.out;
549        //
550        // for (int i = 0; i < comparisonGroupList.size(); i++) {
551        // ps.println("Analysis of \"" + comparisonGroupList.get(i).getDescription() + "\" vs. \""
552        // + comparisonGroupList.get(goldStandardIndex).getDescription() + "\"");
553        // Map<String, PRFStats> scoreMap = scores.get(i);
554        //
555        // Set<String> analysisTypes = scoreMap.keySet();
556        // for (String analysis : analysisTypes) {
557        // scoreMap.get(analysis).printStats(ps);
558        // }
559        // }
560        // }
561    
562        private void note(String message) {
563            System.err.println("NOTE -- AnnotatorComparator: " + message);
564        }
565    
566        private void error(String message) {
567            System.err.println("ERROR -- AnnotatorComparator: " + message);
568        }
569    
570        private void warn(String message) {
571            System.err.println("WARNING -- AnnotatorComparator: " + message);
572        }
573    
574        private void debug(String message) {
575            if (DEBUG) {
576                System.err.println("DEBUG -- AnnotatorComparator: " + message);
577            }
578        }
579    }
```
